# Differential Placing of Flexion Creases Contributes to Sex Differences in the Second-to-Fourth Digit Ratio (2D:4D)

**DOI:** 10.3389/fendo.2019.00537

**Published:** 2019-08-02

**Authors:** Sanjay Kumar, Martin Voracek, Maharaj Singh

**Affiliations:** ^1^Department of Psychology, D.A.V. College, Muzaffarnagar, India; ^2^Department of Basic Psychological Research and Research Methods, Faculty of Psychology, University of Vienna, Vienna, Austria; ^3^Department of Research and Graduate Studies, School of Dentistry, Marquette University, Milwaukee, WI, United States

**Keywords:** digit ratio (2D:4D), flexion creases, fingertip, dorsal finger length, sex differences, prenatal testosterone

## Abstract

The present research examined whether differential placing of the basal flexion creases contributes to the occurrence of sex differences in digit ratio (2D:4D) derived from palmar digit lengths. The ratio of palmar-to-dorsal digit length, a measure of the placing of the basal flexion crease in the finger, was derived for the digits 2 and 4 of the right hand in two independent samples (Study I: *N* = 100; Study II: *N* = 200), in accordance with discovery-replication sample approach. The results show that men have lower palmar-to-dorsal digit ratios than women, and this effect is significantly stronger for digit 2 than for digit 4. Thus, the present study supports the likelihood that differential placing of flexion creases in the digits contributes to the occurrence of sex differences in palmar 2D:4D. In addition (Study II), the measurement procedure assessing the placing of flexion creases was validated. In conclusion, this evidence highlights potential conceptual shortcomings and technical limitations in the measurement conventions and methods currently employed in the field of 2D:4D research.

## Introduction

Studies have reported that sex differences in digit ratio (2D:4D) relate to prenatal sex-hormone levels (1, 2). Because similar sex effects occur in 2D:4D ratios derived either from X-rayed phalangeal bone lengths (henceforth, bone; 3–6) or from palmar digit lengths (1–3), both palmar and bone digit ratios may serve as putative markers for prenatal sex-hormone levels. However, different sets of tissues comprise palmar (fingertip fat and the placing of flexion creases) and bone digit lengths, and different tissues may have differential hormonal sensitivities (as discussed below). Therefore, subtle, but relevant, differences may exist between bone and palmar digit ratios.

Indeed, the absolute values of bone and palmar 2D:4D ratios are quite different (4). Moreover, presumed correlates of prenatal androgens, like enhanced sports performance, fitness, and fertility, seem more consistently related to palmar 2D:4D (5–10), whereas less or not so with the bone 2D:4D ratio [(11, 12), exception: (13)], even in large-sample studies. Similarly, individuals affected with conditions due to very high prenatal testosterone exposure, such as congenital adrenal hyperplasia (14, 15), have lower palmar 2D:4D ratios than controls, whilst no such difference was observed in one study which measured bone 2D:4D (16).

Experimental evidence shows that the proportionate activity of androgen receptors to estrogen receptors-α in digit 4 induces sex effects in bone 2D:4D ratio in rats (17). A polymorphism in the estrogen receptor-α gene accounts for 11% of 2D:4D variation in birds (18), and similar effects are seen in humans (19). Moreover, prenatal testosterone injection produces male-typical changes in dermatoglyphics (20), as well as in palmar digit length, but not in bone digit length, and this effect occurs in digit 2, whereas not in digit 4 (21). Additionally, human epidermal tissues only have androgen receptors and no estrogen receptors-α (22). Because a conserved gene program regulates finger development (23), such studies support the likelihood that bone and dermatoglyphic tissues in fingers may have different sex-hormone sensitivity.

Consistent with the above studies, Manning et al. (24), in a study involving measurements from the same hands, found that sex differences occur in palmar 2D:4D, whereas not in bone 2D:4D. Similarly, a right-hand favor in the occurrence of sex differences is consistently reported for palmar 2D:4D [for a meta-analysis, see (25)], but not for bone 2D:4D (4, 26–29). Because soft tissues (fingertip fat and the placing of flexion creases) are additional components of palmar digit length, these may well have a role in the occurrence of differential sex effects in bone vs. palmar digit ratios. However, studies have shown that fingertip fat is not a factor of sex effects in palmar 2D:4D (4, 26, 30). Therefore, the placing of flexion creases may be the accountable factor.

As of yet, only a few studies have investigated the placing of flexion creases. Studies have reported a stronger sexual dimorphism in digit index (i.e., the ratio of palmar digit 3 length by hand length, which yields lower values in men; 31), which may be a measure of the placing of flexion creases. Thus, although no prior study has studied the differential occurrence of sex effects in the placing of flexion creases in different digits, the account of Kulaksiz and Gozil (31) lends some support to this idea. Moreover, a recent study has supported the likelihood that the placing of flexion creases is a contributing factor in the occurrence of sex differences in digit ratios (32).

To determine the placing of flexion creases, in the present study we measured dorsal and palmar digit lengths and calculated palmar-to-dorsal digit ratios. Because fingertip fat and bone digit length are the common components in dorsal and palmar digit lengths, palmar-to-dorsal digit ratio may serve as a measure of the placing of basal flexion creases. Moreover, because we can measure palmar-to-dorsal digit ratios for different digits, one advantage is that we may derive information about the placing of flexion creases in different digits. Therefore, we measured palmar-to-dorsal digit ratios for digit 2 as well as for digit 4 (in right hands) and tested the following hypotheses. Firstly, men have lower palmar-to-dorsal digit ratio, as compared to women. Secondly, the occurrence of sex differences in palmar-to-dorsal digit ratios differs between digit 2 and digit 4. We tested these hypotheses in two independent samples of adults, according to a discovery-replication sample approach. That is, the findings of the initial (discovery) sample are again addressed in another independent (replication) sample, with the purpose of establishing the robustness and replicability of the phenomena under study.

In addition, in Study II we also attempted to establish the validity of the measurement procedure of the palmar-to-dorsal digit ratios by measuring dorsal digit length via two different procedures, namely one already established (the hand-on-table method; 32) and the other one being a novel approach (the hand-in-air method). These two measurement methods essentially differ with regards to the factor of pressing of fingers against a surface (which is inherent with the hand-on-table method, whereas not so with the hand-in-air method). Because pressing of fingers does not occur in the measurement procedure of palmar digit length, a comparison of observed effects in the two palmar-to-dorsal digit ratios, as derived from two different procedures of measuring digit length dorsally (involving pressing of fingers in one but not in other), may serve to clarify possible measurement-method effects due to pressing of fingers against a surface.

## Materials and Methods

### Study I

#### Participants

**Figure 1:**
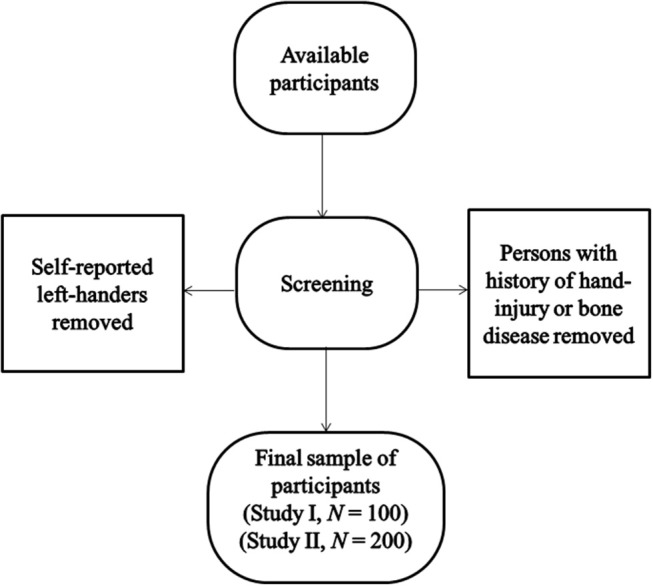
Flow chart of the scheme of selection of participants.

An availability based sample of 100 college students (age: *M* = 23.2 years, *SD* = 5.8; men: *n* = 50, women: *n* = 50) was collected at a college in Muzaffarnagar city, Western Uttar Pradesh, India. Self-reported left hand preference and the history of hand injury or bone disorders were the exclusion criteria (see Figure 1). Similar to Study II, Study I was approved by the D.A.V. College Ethics Committee; participants provided written informed consent prior to the measurements; and all statistical analyses were done with SPSS version 17.0 software.

#### Measurement of Digit Lengths

**Figure 2:**
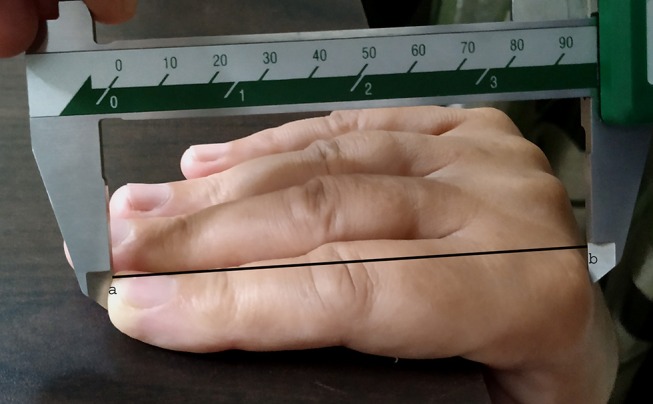
Image showing the measurement method for dorsal digit length through the hand-on-table (HT) procedure. Distance between “a” and “b” represents the dorsal digit length.

**Figure 3:**
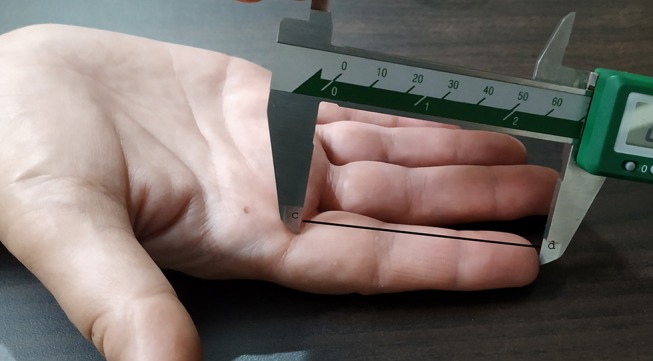
Image showing the measurement method of palmar digit length. Distance between “c” and “d” represents the palmar digit length.

One author (SK) measured dorsal and palmar lengths for digit 2 and digit 4 in the right hand, using vernier calipers with an accuracy level of 0.1 mm. The dorsal digit (dD) length measures the distance between the fingertip and the base of the proximal dorsal phalanx. The investigator asked participants to put their right hand on the edge of a smooth table, with fingers making an angle of 90° to the palm, following the procedure of Kumar et al. [(32); see Figure 2]. The palmar digit (pD) length measures the distance between the fingertip and the basal crease of the proximal palmar phalanx (Figure 3). Participants kept their hands flat, with the palmar side up, on a smooth table during the measurements. We calculated palmar-to-dorsal ratios for digit 2 (2p:2d ratio), as well as for digit 4 (4p:4d ratio; for data, see Supplementary Material).

#### Reliability of Digit Measures

To assess measurement repeatability, we took repeated measurements of dorsal and palmar lengths in digit 2 and in digit 4 from 34 hands (14 men and 20 women). Applying a mixed-effects model of intra-class correlation coefficients (ICCs), with absolute-agreement definition (33), high ICCs (*p* < 0.001) were found for the repeated measurements of digit lengths and digit ratios (2dD length: *ICC* = 0.997; 4dD length: 0.997; 2pD length: 0.992; 4pD length: 0.984; 2p:2d ratio: 0.949, and 4p:4d ratio: 0.893). The relative technical error of measurement (*rTEM*), a measure of error magnitude between repeated measurements, expressed as percentage of the size of the measured variable, also was small for digit lengths (2dD length: 0.85%; 4dD length: 0.91%; 2pD length: 1.46%; 4pD length: 2.34%) and digit ratios (2p:2d ratio: 1.58%, and 4p:4d ratio: 2.44%). Thus, the measurements of digit length and digit ratios had high repeatability.

### Study II

#### Participants

A new sample of 200 right-handed college students (age: *M* = 19.02, *SD* = 1.93, range = 18–24 years; men: *n* = 90, women: *n* = 110), with procedure similar to that followed in the Study I (Figure 1), was collected at a college in Muzaffarnagar.

#### Measurement of Digit Lengths

**Figure 4:**
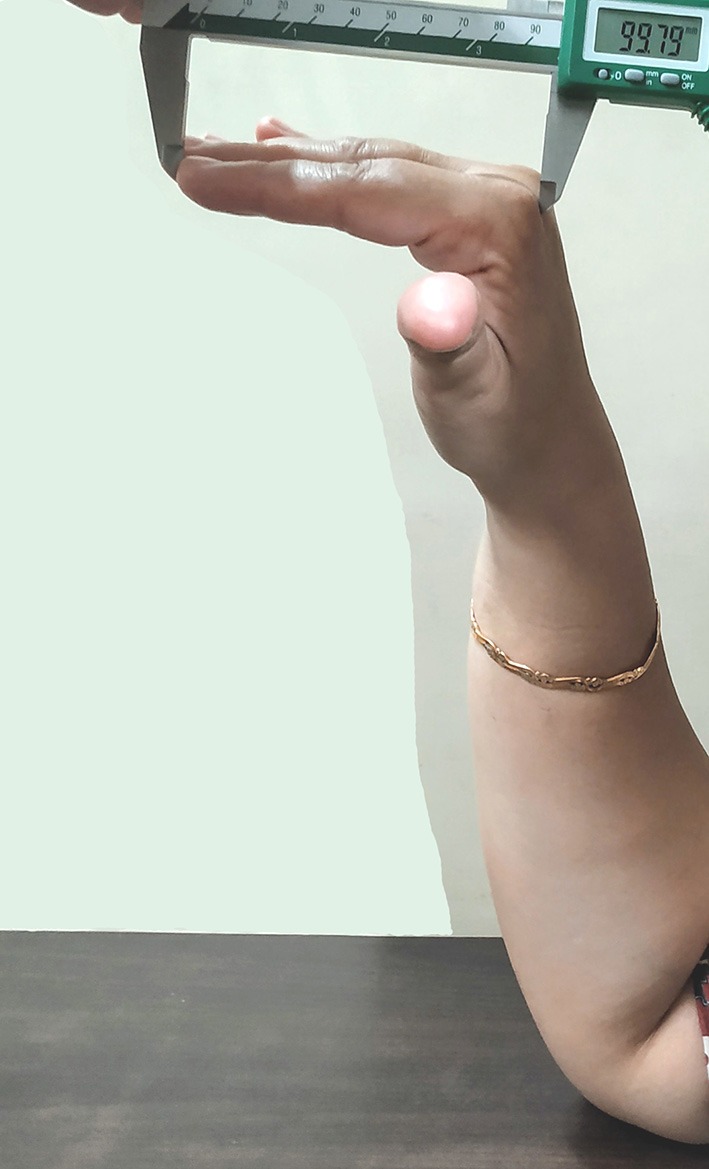
Image showing the measurement method of dorsal digit length through the hand-in-air (HA) procedure.

One investigator (SK) measured dorsal digit length twice, using the two different procedures, and also measured palmar digit length in right-hand digits 2 and 4, using vernier calipers with an accuracy level of 0.01 mm. The measurement procedure for palmar digit length, as well as for one of the dorsal digit lengths (hand-on-table, HT), was the same as in the Study I. However, for the measurement of dorsal digit length via the hand-in-air (HA) procedure, participants were asked to keep their elbow on the table and hold their hand in the air, in such a posture that fingers were straight and parallel to the horizontal surface, whilst making an angle of 90° to the palm (see Figure 4). In some cases, the investigator helped participants to maintain the correct posture, gave demonstrations, as well as instructed them to “freeze” (keep still) the hand in the correct posture, until measurements were done. Two palmar-to-dorsal digit ratios were calculated for digits 2 and 4 each, namely 2p:2d^HT^, 4p:4d^HT^, 2p:2d^HA^, and 4p: 4d^HA^ (for data, see Supplementary Material).

#### Reliability of Digit Measures

To assess the repeatability of measurements, we took repeated measurements of each of the two dorsal and the one palmar digit length in digit 2, as well as in digit 4, in 30 participants (11 men and 19 women). In a mixed-effects model of ICCs (with absolute-agreement definition), high ICCs (*p* < 0.001) were observed for the repeated measurements of digit lengths (2dD^HT^ length: *ICC* = 0.996; 2dD^HA^ length: 0.996; 4dD^HT^ length: 0.991; 4dD^HA^ length: 0.995; 2pD length: 0.986; 4pD length: 0.992) and digit ratios (2p:2d^HT^: *ICC* = 0.86, and 4p:4d^HT^: 0.88; 2p:2d^HA^: 0.90, and 4p:4d^HA^: 0.91). Also, the *rTEM* was small for digit lengths (2dD^HT^: 0.59%; 4dD^HT^: 0.89%; 2dD^HA^: 0.64%; 4dD^HA^: 0.79%; 2pD: 0.87%; 4pD: 1.65%) and digit ratios (2p:2d^HT^: 1.34%; 4p:4d^HT^: 1.16%; 2p:2d^HA^: 1.41%; 4p:4d^HA^: 1.13%). Thus, the measurements of digit lengths and digit ratios, through both procedures, were highly repeatable.

We also assessed inter-observer reliability. Two trained investigators repeated the measurement of the two dorsal digit lengths and the one palmar digit length in digits 2 and 4 in a different set of 20 participants (5 men and 15 women). In a mixed-effects ICC model (with absolute-agreement definition), high ICCs (*p* < 0.001) were observed for the measurements of the two investigators for digit lengths (2dD^HT^ length: *ICC* = 0.956; 2dD^HA^ length: 0.958; 4dD^HT^ length: 0.968; 4dD^HA^ length: 0.967; 2pD length: 0.934; 4pD length: 0.962) and digit ratios (2p:2d^HT^: *ICC* = 0.82; 4p:4d^HT^: 0.80; 2p:2d^HA^: 0.82; 4p:4d^HA^: 0.78). Thus, a sufficiently high inter-observer repeatability was also observed regarding the measurements of digit lengths and digit ratios.

#### Analysis

We conducted a three-way mixed-design analysis of variance, with palmar-to-dorsal digit ratio (for digit 2 vs. for digit 4) and measurement-procedure (HT vs. HA) as the two within-subject factors and participant sex (men vs. women) as the between-subjects factor.

## Results

### Study I

A two-way mixed-design analysis of variance showed significant main effects for the within-subject factor of digit (digit 2 vs. digit 4), *F*_(1, 98)_ = 103, *p* < 0.001, η_p_2 = 0.513, and the between-subjects factor participant sex (male vs. female), *F*_(1, 98)_ = 8.43, *p* = 0.005, η_p_2 = 0.079, as well as an interaction effect of digit by sex, *F*_(1, 98)_ = 12.4, *p* = 0.001, η_p_2 = 0.112, on palmar-to-dorsal digit ratios.

The main effect for digit (*d* = 0.90) was such that palmar-to-dorsal ratios were higher for digit 2 (*M* = 0.721, *SD* = 0.026) than for digit 4 (*M* = 0.699, *SD* = 0.023). Thus, the placing of flexion creases in digit 2 is more proximal than in digit 4. Palmar-to-dorsal digit ratios were lower among men than among women (significant main effect for participant sex). Hence, men present a relatively distal placing of flexion creases (Table 1). Moreover, sex differences in palmar-to-dorsal digit ratios are stronger in digit 2 than in digit 4 (a significant sex by digit interaction effect; Table 1). The effect size of sex differences in the palmar-to-dorsal digit ratio of digit 2 is large (Table 1). Table 2 shows the descriptive statistics of palmar and dorsal digit lengths for men, women, and the total sample.

**Table 1:** Descriptive statistics and sex differences in palmar-to-dorsal digit ratios (Study I).

	**Men**		**Women**		**Cohen's**
	***M***	***SD***	***M***	***SD***	***d***
2p:2d ratio	0.711	0.022	0.731	0.025	−0.85^a^
4p:4d ratio	0.697	0.020	0.701	0.025	−0.18

a *p < 0.001*.

**Table 2:** Descriptive statistics of palmar and dorsal digit lengths (in mm) for men, women, and the total sample (Study I).

	**Men**		**Women**		**Total**	
	***M***	***SD***	***M***	***SD***	***M***	***SD***
Dorsal digit 2 length	101.26	5.95	92.01	4.62	96.63	7.05
Palmar digit 2 length	72.00	4.45	67.27	4.46	69.63	5.03
Dorsal digit 4 length	108.18	5.83	99.12	4.89	103.65	7.03
Palmar digit 4 length	75.36	4.58	69.48	4.45	72.42	5.38

### Study II

Results of the mixed-design analysis of variance showed significant main effects of digit ratio, *F*_(1, 198)_ = 161, *p* < 0.001, η_p_2 = 0.449, and of measurement procedure, *F*_(1, 198)_ = 41.8, *p* < 0.001, η_p_2 = 0.174, along with significant two-way interaction effects of sex by digit ratio, *F*_(1, 198)_ = 8.94, *p* = 0.003, η_p_2 = 0.043, and of measurement procedure by digit ratio, *F*_(1, 198)_ = 7, *p* = 0.009, η_p_2 = 0.034. The three-way interaction effect of measurement procedure by digit ratio by sex was not nominally significant, *F*_(1, 198)_ = 3.63, *p* = 0.06, η_p_2 = 0.018, whereas the main effect for participant sex was significant, *F*_(1, 198)_ = 7.13, *p* = 0.008, η_p_2 = 0.035.

The digit ratio effect (HT procedure: *d* = 0.88; HA procedure: *d* = 0.76) showed that palmar-to-dorsal digit ratios were higher for digit 2 (2p:2d^HT^ ratio: *M* = 0.713, *SD* = 0.024; 2p:2d^HA^ ratio: *M* = 0.709, *SD* = 0.023) than for digit 4 (4p:4d^HT^ ratio: *M* = 0.694, *SD* = 0.019; 4p:4d^HA^ ratio: *M* = 0.693, *SD* = 0.019). That is, for either of the procedures (HT and HA), the placing of flexion creases was more proximal for digit 2 than for digit 4. However, measurements of palmar-to-dorsal digit ratios were higher with the HT procedure than with the HA procedure (i.e., a measurement-procedure effect; Table 3). Because the palmar digit lengths used in the two procedures are the same, this reflects shorter measurements of dorsal digit lengths with the HT procedure than with the HA procedure (see Table 4).

**Table 3:** Descriptive statistics and sex differences in palmar-to-dorsal digit ratios derived through the HT and HA measurement procedures (Study II).

	**Men**		**Women**		**Cohen's**
	***M***	***SD***	***M***	***SD***	***d***
2p:2d^HT^ ratio	0.706	0.024	0.718	0.022	−0.52^a^
4p:4d^HT^ ratio	0.693	0.021	0.695	0.018	−0.10
2p:2d^HA^ ratio	0.704	0.023	0.714	0.021	−0.46^b^
4p:4d^HA^ ratio	0.691	0.021	0.694	0.017	−0.16

a *p < 0.001*.

b *p < 0.01*.

**Table 4:** Descriptive statistics of palmar and dorsal digit lengths (in mm) for men, women, and the total sample (Study II).

	**Men**		**Women**		**Total**	
	***M***	***SD***	***M***	***SD***	***M***	***SD***
Dorsal digit 2 length ^HT^	100.26	5.06	92.03	4.50	95.74	6.28
Dorsal digit 2 length ^HA^	100.56	5.08	92.59	4.72	96.17	6.29
Palmar digit 2 length	70.80	4.46	66.10	3.92	68.21	4.77
Dorsal digit 4 length ^HT^	107.46	5.34	98.26	5.11	102.40	6.94
Dorsal digit 4 length ^HA^	107.71	5.22	98.46	5.02	102.62	6.88
Palmar digit 4 length	74.45	4.56	68.34	4.09	71.09	5.27

**Figure 5:**
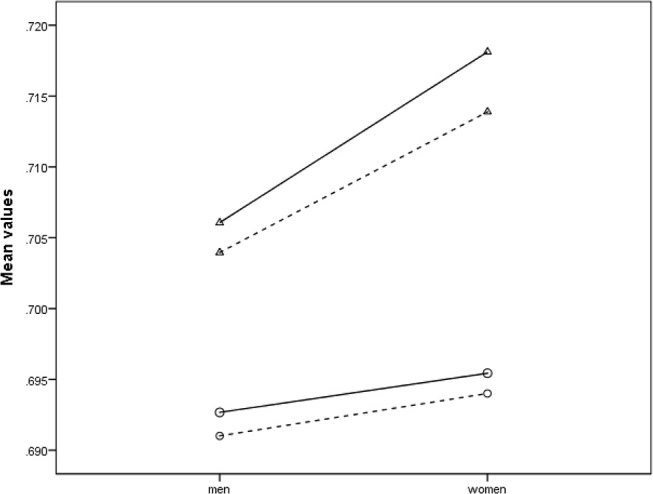
Line graph showing sex differences in palmar-to-dorsal digit ratios of digit 2 (Δ) and digit 4 (O), measured through the HT (__) and the HA (−−−) procedures. (i) Men have lower palmar-to-dorsal digit ratios than women, and this effect is stronger for digit 2 than for digit 4. (ii) The pattern of sex differences observed in digits 2 and 4 is similar for both (HT or HA) procedures. (iii) Digit ratios derived from the HT procedure are higher than those derived from the HA procedure, and, among both men and women, this effect is stronger for digit 2 than for digit 4.

Palmar-to-dorsal digit ratios were higher among women than among men (main effect for participant sex). However, this sex effect occurred more strongly in digit 2 than in digit 4 (sex by digit ratio interaction effect; see Table 3 and Figure 5). Thus, for either of the procedures (HT and HA), the effect of a more distal placing of flexion creases among men (as compared to women) was stronger for digit 2 than for digit 4. Furthermore, effect sizes for observed sex differences in the palmar-to-dorsal digit ratios of digit 2 were intermediate (Table 3).

Additionally, the tendency of higher palmar-to-dorsal digit ratios (i.e., a shorter dorsal digit length) with the HT procedure, as compared to the HA procedure, was stronger for digit 2 than for digit 4 (measurement by digit ratio interaction effect, see Table 3 and Figure 5). However, this also had a relationship with the occurrence of sex differences in palmar-to-dorsal digit ratios (measurement by digit ratio by sex interaction effect; see Figure 5).

## Discussion

### Study I

The results support the likelihood that the differential placing of flexion creases contributes to the occurrence of higher values of palmar 2D:4D ratio, as compared to bone 2D:4D ratio. Studies have shown that genes (34), rather than flexion movements (35), are the likely determinants of the formation of flexion creases. Therefore, the distal placing of flexion creases in digit 4, as compared to in digit 2, may have genetic bases.

Consistent with the first hypothesis, the evidence suggests that the placing of flexion creases is more distal in men than in women. Flexion creases appear between fetal weeks 6 to 13 (36). This is the time window in which testosterone production starts in the gonads of fetuses (37). Studies have reported that prenatal testosterone modulates dermatoglyphic patterns (20, 38, 39), and human epidermal tissues predominantly have androgen receptors (22). Therefore, prenatal testosterone may be a determinant of observed sex effects in the placing of flexion creases.

Moreover, the data at hand support the second hypothesis. Thus, the differential placing of flexion creases likely is a factor in the occurrence of sex differences in palmar 2D:4D ratios. Perhaps prenatal testosterone affects different digits differentially. Various animal experimental studies have shown that prenatal testosterone injection affects digit 2 length, but not digit 4 length [in monkeys: (21); in frogs: (40)]. Because the placing of flexion creases follows a hand-wide developmental plan, which likely is genetically determined, prenatal testosterone may modulate this developmental plan of the hand and may be responsible for differential sex effects in the placing of flexion creases across different digits. Furthermore, the placing of flexion creases in digit 2 may be a stronger indicator of prenatal testosterone levels than the traditionally studied digit ratios [palmer 2D:4D; sexual dimorphism, *d* = 0.35, (25)].

A possible limitation of the present approach is that the procedures of measuring dorsal and palmar digit lengths differ with respect to the pressing of fingers against a surface (present in dorsal, but not palmar, digit-length measurement). Because the fingertip is much fatter in digit 4 than in digit 2 (30), the measurement procedure of digit length (dorsal vs. palmar) may interact with fingertip fat (digit 2 vs. digit 4) and may produce lower palmar-to-dorsal digit ratios for digit 4 than for digit 2. However, because studies have shown that the amount of fingertip fat (of digit 2, as well as of digit 4) is not related to image-based 2D:4D measurements (where digits are pressed against a surface; 30), the pressing of fingers does not seem to be the factor that affects digit ratio measurement. Furthermore, because the amount of fingertip fat is barely sexually differentiated (4, 30), this is unlikely to have effects on the occurrence of sex differences in palmar-to-dorsal digit ratios.

### Study II

The results of Study II supported all the hypotheses and replicated all findings of Study I. This strengthens the likelihood that the placing of flexion creases is more distal in men than in women, and that the differential placing of flexion creases plays some role in the occurrence of sex differences, as well as in the occurrence of comparatively higher values, in palmar 2D:4D ratios.

Additionally, the occurrence of similar effect patterns in the placing of flexion creases, as measured through the HT and HA procedures, provides support for the contention that the pressing of fingertip fat is not likely a confounding factor. In the HT measurement procedure, pressing of fingers against a surface occurs, whereas with the HA procedure, no such pressing occurs. Hence, the current results validate the above reported effects for the placing of flexion creases, as derived from the HT procedure. Put another way, the HT procedure provides valid measurements of the placing of flexion creases.

On the other hand, despite convergent findings, there are some differences in digit lengths, when measured through the HT vs. HA procedures. Thus, the less than perfect similarity could be seen as a limitation on the above-reported validation results. However, this is not likely the case. For example, assuming that the pressing of fingertip fat is a confounding factor, dorsal digit lengths should be longer, when measured with the HT procedure (where fingers are pressed) than when measured with the HA procedure. Furthermore, because the fingertip of digit 4 is more fatty than the one of digit 2 (30), unlike the HA procedure, with the HT procedure dorsal digit length should be extra-long for digit 4, in comparison with digit 2. However, the present results yield just the opposite pattern, i.e., dorsal digit lengths are longer with the HA procedure than with the HT procedure, and this observed difference is larger for digit 2 than for digit 4. Hence, despite some differences between the HT and HA procedures, the present evidence does not support the possibility that the pressing of fingertip fat would be a confounding factor in the placing of flexion creases.

Moreover, in the HA procedure the fingers are less likely to be straight (with no support) and can easily attain a 90° angle (because of free posture) between fingers and palm, as compared to the HT procedure, in which fingers are straightened by a flat surface and the posture is constrained by the table edge. Because imperfect straightening of fingers and easy attainment of a 90° angle between finger and palm favors shorter digit-length measurements, the HA procedure may yield lower mean values of digit length than the HT procedure. However, the directionally opposite results (namely, higher mean dorsal digit lengths with the HT than with the HA procedure) do not support this idea.

Therefore, the attainment of a perfect 90° angle in hand posture and the straightness of fingers likely are not significant factors.

The occurrence of longer dorsal digit lengths through the HA procedure, as compared to the HT procedure, is unexpected and requires an explanation. A difference between the HA and HT procedures is that in the former, fingers are deliberately straightened, whereas in the latter, fingers are straightened with the support of the smooth surface of the table (a detail which we failed to contemplate when the study was designed). Because fingers have very little muscles, the stretching of muscles during straightening of fingers may have no effect. Instead, there is a web of veins and capillaries in the fingertip, and this web is likely to be more dense in digit 2 than in digit 4 (41, 42). Therefore, with deliberate straightening of fingers, more blood may flow into the fingertips and, in particular, more so into the tip of digit 2 than into the tip of digit 4. Although we are not aware of specific studies which would support the obvious expectation that increased inflow of blood inflates the fingertips, this seems to be the most likely cause of the longer dorsal digit lengths occurring in the measurements with the HA procedure, as compared to the HT procedure.

Furthermore, because deliberate straightening leads to digit elongation, the present evidence identifies this as a potential confounding factor that would require control during the measurement of digit lengths and digit ratios. Relatedly, it is emphasized that digit elongation through the HA procedure does not affect the occurrence of sex effects in the placing of flexion creases, and therefore the likelihood that the differential placing of flexion creases contributes to the sex effect in the palmar 2D:4D ratio is supported. Nevertheless, digit elongation interferes with determining the actual positions of the placing of flexion creases. Hence, devising procedures that neither involves pressing, nor deliberate straightening of fingers is desirable and would be a favorable and fruitful focus of future investigations.

### General Discussion

The present two studies, thus, support the following likelihoods related to the placing of flexion creases. The differential placing of flexion creases contributes to the occurrence of higher values of palmar 2D:4D ratio, as compared to bone 2D:4D ratio; the placing of flexion creases is more distal in men than in women; the differential placing of flexion creases likely is a factor in the occurrence of sex differences in palmar 2D:4D ratios, and; lastly, the pressing of fingers conceivably may not confound effects of digit-ratio measurement.

Moreover, apart from the differential placing of flexion creases, the relative length of finger and hand bones are additional factors involved in the occurrence of sex effects in the 2D:4D ratio (17). Therefore, the present studies also highlight the need to revisit, in general, the findings of earlier studies on the palmar 2D:4D ratio and, in particular, studies reporting that the effects occur for palmar digit ratios, but not for bone digit ratios. Candidate studies to be revisited (as reviewed above), in the light of the current evidence, thus are reports of presumed sex-hormone effects (for example, fitness, fertility, and congenital adrenal hyperplasia), as observed for palmar digit ratios, but not observed for bone digit ratios, as well as studies reporting the sex effect for palmar 2D:4D ratio only for the right hand, but for bone 2D:4D ratio reporting similar effects in both hands. In addition, because different hormonal determinants may be involved, the simultaneous study of the placing of flexion creases and bone digit length ratios in a hand may provide a richer model for understanding such effects.

Although, by pursuing a discovery-replication approach, we sought to replicate our initial findings (from Study I) internally (via Study II), thereby validating the novel measurement procedures introduced here, several limitations remain to be addressed by future inquiries along these lines. The findings originate from a single study locale. It would therefore be beneficial to replicate them in other geographic regions. Moreover, the findings of both Studies I and II are based on samples of young, and presumably healthy, adults, and thus the generalizability of the findings to other specific age brackets (younger samples: toddlers, children, and adolescents; as well as more mature samples: middle age and the elderly) remains to be tested. As well, it would be profitably to assess the generalizability of findings to various clinical sample groups that would be of interest to 2D:4D research.

In conclusion, the results are suggestive for men having lower palmar-to-dorsal digit ratios than women, with this effect being stronger for digit 2 than for digit 4. This lends support to the idea that the differential placing of flexion creases in the digits contributes to the occurrence of sex differences in palmar 2D:4D. On the whole, this evidence highlights potential conceptual shortcomings and technical limitations in measurement conventions and the measurement methods currently employed in the 2D:4D research literature.

## Ethics Statement

This study was carried out in accordance with the recommendations of ‘D.A.V. College Ethics Committee’ with written informed consent from all subjects. All subjects gave written informed consent in accordance with the Declaration of Helsinki. The protocol was approved by the ‘D.A.V. College Ethics Committee’.

## Author Contributions

SK and MV contributed to the planning of the study, analysis of the data, and intellectual input. MS also contributed in analysis and interpretation of the results.

### Conflict of Interest Statement

The authors declare that the research was conducted in the absence of any commercial or financial relationships that could be construed as a potential conflict of interest.
